# Egg white hydrolysate shows insulin mimetic and sensitizing effects in 3T3-F442A pre-adipocytes

**DOI:** 10.1371/journal.pone.0185653

**Published:** 2017-10-03

**Authors:** Forough Jahandideh, Subhadeep Chakrabarti, Sandra T. Davidge, Jianping Wu

**Affiliations:** 1 Department of Agricultural, Food and Nutritional Science, University of Alberta, Edmonton, Alberta, Canada; 2 Cardiovascular Research Centre, University of Alberta, Edmonton, Alberta, Canada; 3 Department of Obstetrics and Gynecology, University of Alberta, Edmonton, Alberta, Canada; 4 Department of Physiology, University of Alberta, Edmonton, Alberta, Canada; 5 Women and Children’s Health Research Institute, University of Alberta, Edmonton, Alberta, Canada; Tokyo University of Agriculture, JAPAN

## Abstract

Insulin resistance and inflammation in adipose tissue is a key mechanism underlying metabolic syndrome, a growing health problem characterized by diabetes, obesity and hypertension. Previous work from our research group has demonstrated the potential of egg white ovotransferrin derived bioactive peptides against hypertension, oxidative stress and inflammation *in vitro* and *in vivo*. Egg white hydrolysate (EWH) has also shown anti-hypertensive effects in spontaneously hypertensive rats. Given the interplay among hypertension, inflammation, oxidative stress and metabolic syndrome, the objective of the study was to test the EWH on differentiation, insulin signaling and inflammatory responses in 3T3-F442A pre-adipocytes. Our study suggested that EWH could promote adipocyte differentiation as shown by increased lipid accumulation, increased release of adiponectin and upregulation of peroxisome proliferator associated receptor gamma (PPARγ) and CCAAT/ enhancer binding protein alpha (C/EBP-α). In addition to enhanced insulin effects on the upregulation of protein kinase B/Akt phosphorylation, EWH treatment increased extracellular signal regulated kinase 1/2 (ERK1/2) phosphorylation to a level similar to that of insulin, indicating insulin sensitizing and mimetic properties of the EWH. EWH further attenuated cytokine induced inflammatory marker; cyclooxygenase -2 (COX-2) by 48.78%, possibly through the AP-1 pathway by down regulating c-Jun phosphorylation in adipocytes. Given the critical role of adipose in the pathogenesis of insulin resistance and metabolic syndrome, EWH may have potential applications in the prevention and management of metabolic syndrome and its complications.

## Introduction

Metabolic syndrome, a combination of several abnormalities that increase the risk for type II diabetes and atherosclerosis is global health problem of growing concern [1–3]. It consists of atherogenic dyslipidemia (elevated triglycerides and low high-density lipoproteins), hypertension, glucose intolerance, and proinflammatory states [4].

Hypertension and insulin resistance are the key features of metabolic syndrome. Renin angiotensin system (RAS), the classical pathway for controlling blood pressure and fluid balance, has also a role in the pathogenesis of metabolic syndrome [5]. In the RAS, angiotensin converting enzyme (ACE) plays a critical role in the formation of angiotensin II (Ang II), the primary active peptide of this system which increases blood pressure by enhancing vascular constriction. RAS blockade by ACE-inhibitors or angiotensin receptor blockers beneficially affects insulin sensitivity and prevents the development of diabetes [6,7]. Given the role of RAS impairments in the pathogenesis of hypertension and metabolic syndrome, there is much interest in developing novel therapies that can target the common pathologies to hypertension and insulin resistance in more complicated disease conditions [2,8,9].

Insulin is essential for normal metabolic functions of various tissues in the body [10]. Adipose tissue with a central role in lipid and glucose metabolism is a key target of insulin [11]. Insulin promotes differentiation of pre-adipocytes into mature adipocytes; a process accompanied by incorporation of lipid droplets and upregulation of immunomodulatory proteins like peroxisome proliferator associated receptor gamma (PPARγ) [12]. Collectively, insulin actions on adipose tissue appear to be beneficial and anti-inflammatory in nature. Under metabolic syndrome, insulin signaling in adipose tissue is perturbed, associated with insulin resistance and chronic inflammation [9,13–16]. As such, there is significant interest in developing therapeutic agents to improve insulin signaling in adipocytes, either by insulin sensitizing agents or through agents mimicking insulin actions [17,18]. The insulin sensitizing drugs thiazolidinediones (TZDs) enhance adipocyte differentiation. This increases lipid partitioning into adipocytes and decreases circulating, hepatic, and intramuscular triglycerides thus enhances insulin sensitivity [19].

Use of pharmacological drugs for controlling different complications of metabolic syndrome is associated with significant risk of side-effects especially when lifelong therapy is required [20]. Not surprisingly, there is growing interest in developing naturally based products to attenuate insulin resistance as safer alternatives. Food derived products are valuable sources of novel therapeutic agents which are generally perceived as safer options compared to synthetic pharmacological drugs [21]. Several food proteins derived hydrolysates and peptides have undergone evaluation for therapeutic usage in metabolic disorders [22, 23].

Egg is a valuable source of dietary proteins. In addition to the nutritional value, egg proteins are also a source for peptides with myriad bioactive properties [24], including ACE inhibition [25–27]. Previous work from our research group has demonstrated the potential of egg white protein ovotransferrin derived bioactive peptides against hypertension, oxidative stress and inflammation *in vitro* and *in vivo* [25,28,29]. Moreover, we have recently reported the effects of egg white hydrolysate (EWH) on reducing blood pressure in hypertensive rats [30]. EWH significantly reduced blood pressure through modulating RAS components, reducing nitrosative stress and enhancing vascular relaxation [30]. While some features of metabolic syndrome such as inflammation and hypertension appear amenable to treatment with egg white protein derivatives [25,28–31], their actions on adipocyte functions have remained largely unknown.

Given the interplay among hypertension, inflammation, and metabolic syndrome, the objective of the study was to test the effect of EWH on differentiation, insulin signaling and inflammatory responses in 3T3-F442A pre-adipocytes. The findings of this study indicate insulin mimetic and insulin sensitizing as well as anti-inflammatory actions of EWH in adipocytes which may potentially prevent or alleviate the complications of metabolic syndrome.

## Material and methods

### Reagents

Pasteurized liquid egg white was purchased from Egg Processing Innovation Cooperative (Lethbridge, Alberta, Canada). Dulbecco’s phosphate buffered saline (PBS), LipidTox dye and dithiothreitol (DTT) were all bought from Sigma Aldrich (St Louis, MO, USA). Dulbecco’s modified Eagle medium (DMEM) and fetal bovine serum (FBS) were from Gibco/ Invitrogen (Carlsbad, CA, USA). The murine tumor necrosis factor alpha (TNF-α) was obtained from Peprotech (Rocky Hill, NJ, USA). Triton-X-100 was from VWR International (West Chester, PA, USA). Type 1 Collagenase used for cell splitting was from Worthington Biochemical Corporation (Lakewood, NJ, USA). Thermoase PC10F (from *Bacillus thermoproteolyticus* Var. Rokko) was purchased from Amano Enzyme Inc. (Nagoya, Japan). Pepsin (from porcine stomach, 10000 units / mg) was purchased from American Laboratories Inc. (Omaha, NE, USA).

### Preparation of egg white hydrolysate (EWH)

Hydrolysis of egg white was carried out according to our previous method with slight modifications [32]. Briefly, liquid egg white was diluted with water at a ratio of 1:1 (v/v) to obtain a solution with 5% protein solid. After adjusting the pH to 8.0 with 2 M NaOH solution, and the temperature to 65°C, thermoase (0.1%, w/w) was added and protein digestion was carried out for 90 min. The enzyme was then inactivated by adjusting pH to 2.5 for pepsin digestion. The mixture was further hydrolyzed at 55°C by 1% pepsin for 180 min. The reaction was terminated by heating the solution at 95°C for 15 min and the hydrolysate was centrifuged and then condensed to obtain approximately 10% solid. The hydrolysate was then spray dried and the powder was collected and stored -20°C for further experiments. EWH was desalted with 50% acetonitrile/deionized water using Sep-Pak C18 cartridges (product #: WAT043345, Waters, Ontario, Canada) to remove salts in the hydrolysate for using in cell experiments.

### Cell culture & differentiation

The murine pre-adipocyte cell line 3T3-F442A (Sigma Aldrich; Cat# 00070654) was used. Cell culture method is similar to our previous study [33]. The cells were obtained in passage 8, thawed and expanded in culture using DMEM supplemented with 10% FBS (heat-inactivated) and antibiotics. The cells were grown in T-25 flasks to confluence prior to sub-culture in gelatin-coated 48 well plates. All studies were performed using cells in passages 11–37.

To determine the ability of EWH to induce adipogenic differentiation, the cells (grown in 48 well plates) were incubated in standard culture medium (DMEM + 10% FBS + antibiotics) in the presence of EWH or insulin for 72 h without changing the medium. Adipogenic changes were determined by the appearance of intracellular lipid droplets (as shown by LipidTox staining), upregulation of PPARγ and CCAAT/ enhancer binding protein alpha (C/EBP-α) (determined by western blot) and release of adiponectin (measured by ELISA). Insulin (10 μg/mL) was used only as a positive control for inducing differentiation.

S961/Insulin Receptor Antagonist (cat#051–86, Phoenix pharmaceuticals Inc. USA) was used at the concentration of 200 nM to investigate the involvement of insulin receptor for the potential effects of EWH on insulin signaling.

For inflammation studies, confluent monolayers of cells were treated with/ without EWH for 48 h followed by administration of murine TNF-α (24 hrs for COX-2, 15 minutes for cell signaling experiment).

### Intracellular lipid staining

Intracellular lipid accumulation, a marker for adipogenic differentiation, was determined by LipidTox staining as described in our previous study [33]. Briefly, the cells were treated for 72 h with EWH or insulin (positive control), fixed and stained with LipidTox (1:250 in phosphate buffered saline) and counter-stained with the nuclear dye Hoechst 33342. The cells were then visualized under an Olympus IX81 fluorescent microscope (Carson Scientific Imaging Group; Markham, Ontario, Canada). Images were obtained and analyzed using the Metamorph imaging software (Molecular Devices, Sunnyvale, CA) and presented at (200X) magnification. A control image from a group of cells without LipidTox was used to detect any nonspecific fluorescence. The images were then quantified by subtracting the background fluorescence of the control image, so only fluorescence from the lipid-specific staining was visible. The cell nuclei were stained by the DNA stain Hoechst3342. The fluorescence intensity was then measured for quantitative analysis and quantified as mean intensity per cell (MFI/cell) and expressed as % of untreated cells.

### Adiponectin measurement

The culture media from untreated and EWH (or insulin) treated cells were centrifuged (10,000 g for 10 min at 4°C) to yield cell-free supernatants which were stored at -80°C until time of the assay. These supernatants were thawed and used in the Mouse Adiponectin DuoSet ELISA kit (R&D Systems; Minneapolis, MN, USA) following the manufacturer’s instructions. Data were normalized to supernatants from the untreated cells.

### Western blotting

Western blotting was done on 3T3-F442A cell lysates prepared at the end of experimental procedures as described in our previous studies [33,34]. Protein bands for C/EBP-α (rabbit polyclonal antibody from Cell Signaling Technology, Boston, MA, USA, cat# 2295), PPARγ (rabbit polyclonal antibody from Cell Signaling Technology, cat# 2430), phospho-Akt (rabbit polyclonal antibody from Cell Signaling Technology, cat #9271), Akt (mouse monoclonal from Santa Cruz, cat#sc-81434), phospho-ERK1/2 (rabbit polyclonal antibody from Cell Signaling Technology, cat#9101), ERK1/2 (mouse monoclonal antibody from Cell Signaling Technology, cat#4696), phospho-IRS-1 (rabbit polyclonal antibody from Cell Signaling Technology, cat#3070), IRS-1 (mouse monoclonal antibody from Santa Cruz Biotechnology, Santa Cruz, CA, USA, cat# sc-8038), phospho-p65 (rabbit polyclonal antibody from Santa Cruz Biotechnology, cat# sc-3033) and p65 (mouse monoclonal antibody from Santa Cruz Biotechnology, cat# sc-8008) were normalized to α-tubulin (rabbit polyclonal antibody from Abcam, Cambridge, MA, cat# ab15246). Anti-tubulin was used at 0.4 μg/ml, while all other antibodies were used at 0.5–1 μg/ml. Goat anti-rabbit and Donkey anti-mouse conjugated secondary antibodies were purchased from Li-cor Biosciences (Lincoln, NB). The protein bands were detected by a Li-cor Odyssey BioImager and quantified by densitometry using corresponding software Odyssey v3.0 (Li-cor). Cell lysates from untreated cells were loaded on every gel and all data were expressed as % of the corresponding untreated control.

### Statistical analysis

All data are expressed as mean±SEM (standard error of mean) of 4–8 independent experiments. Data were analyzed by one-way analysis of variance (ANOVA) with an appropriate post-hoc test (Dunnett’s test for comparison to control group; Tukey’s test for multiple comparisons). For studying interactions between 2 independent variables (e.g. EWH and insulin), two-way ANOVA was used. A repeated measures test was used when applicable. The PRISM 6 statistical software (GraphPad Software, San Diego, CA) was used for the analyses. A value of p< 0.05 was considered significant.

## Results

### EWH treatment induces adipogenic differentiation in 3T3-F442A cells

**Fig 1:**
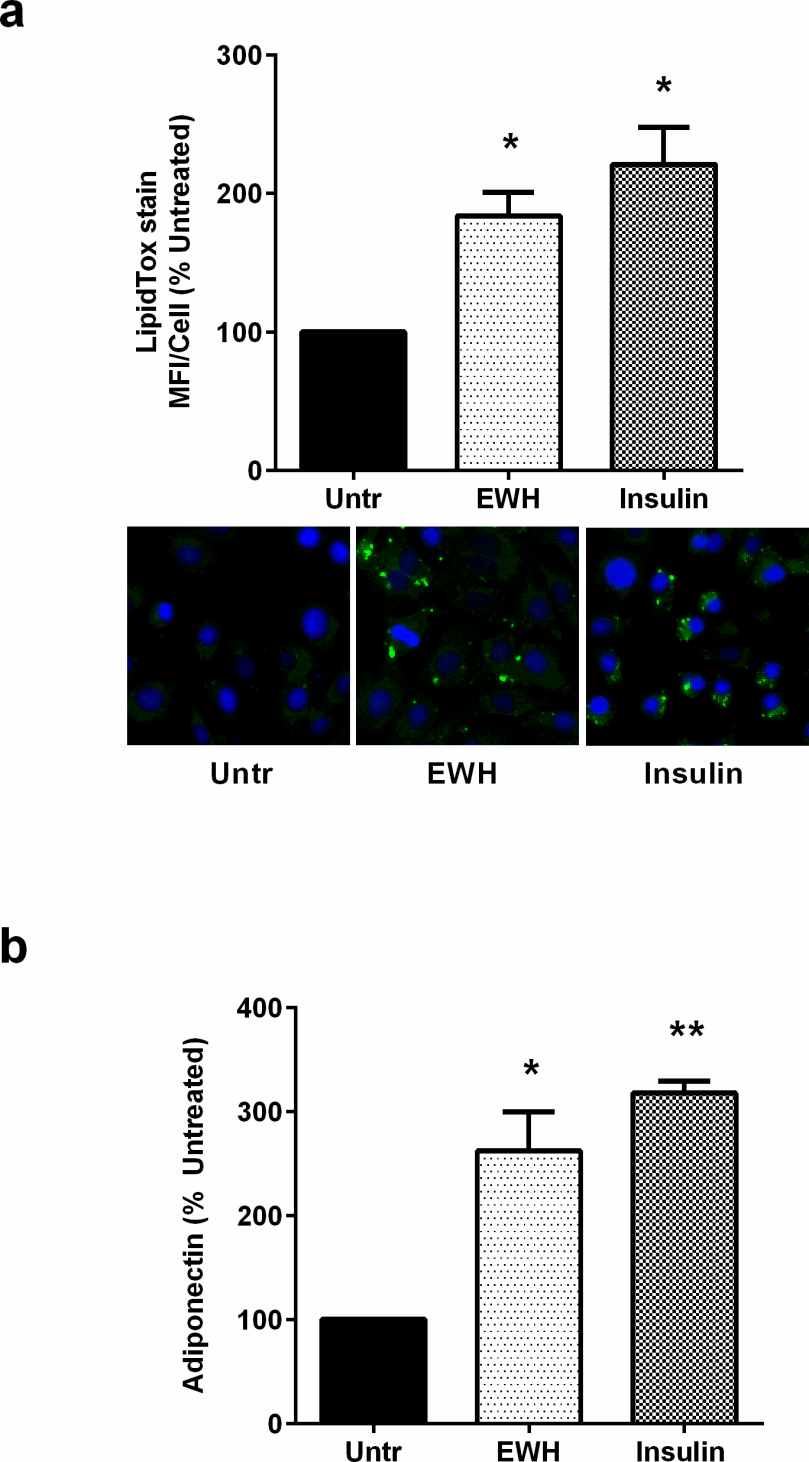
EWH treatment induces adipogenic differentiation in 3T3-F442A cells. 3T3-F442A cells were incubated with EWH (5 mg/mL) or insulin (positive control; 10 μg/mL) for 72 hrs. (a) Following incubation, the cells were fixed and stained with the neutral lipid-specific dye LipidTox (green), the nuclear stain Hoechst3342 (blue) and visualized under fluorescence microscopy. A set of representative images are shown. (b) The cell-free culture supernatants were collected and analyzed by ELISA to determine adiponectin levels. Data are presented as mean±SEM of 4–5 independent experiments. * and ** indicate p<0.05 and p<0.01 respectively compared to the untreated control (Untr).

Adipogenic differentiation in pre-adipocytes is characterized by increases in intracellular lipid droplets and release of adiponectin [35,36]. Treating 3T3-F442A cells with EWH for 72 hrs resulted in increased intracellular lipid accumulation as determined by LipidTox staining (Fig 1A). These changes were accompanied by higher levels of adiponectin released into the culture medium, further demonstrating the pro-differentiation properties of EWH (Fig 1B). Interestingly, both effects induced by EWH were similar in magnitude to those of insulin, suggesting that the beneficial effects of EWH could be comparable to the physiological effects of insulin.

### EWH upregulates markers of adipocyte differentiation in 3T3-F442A cells

**Fig 2:**
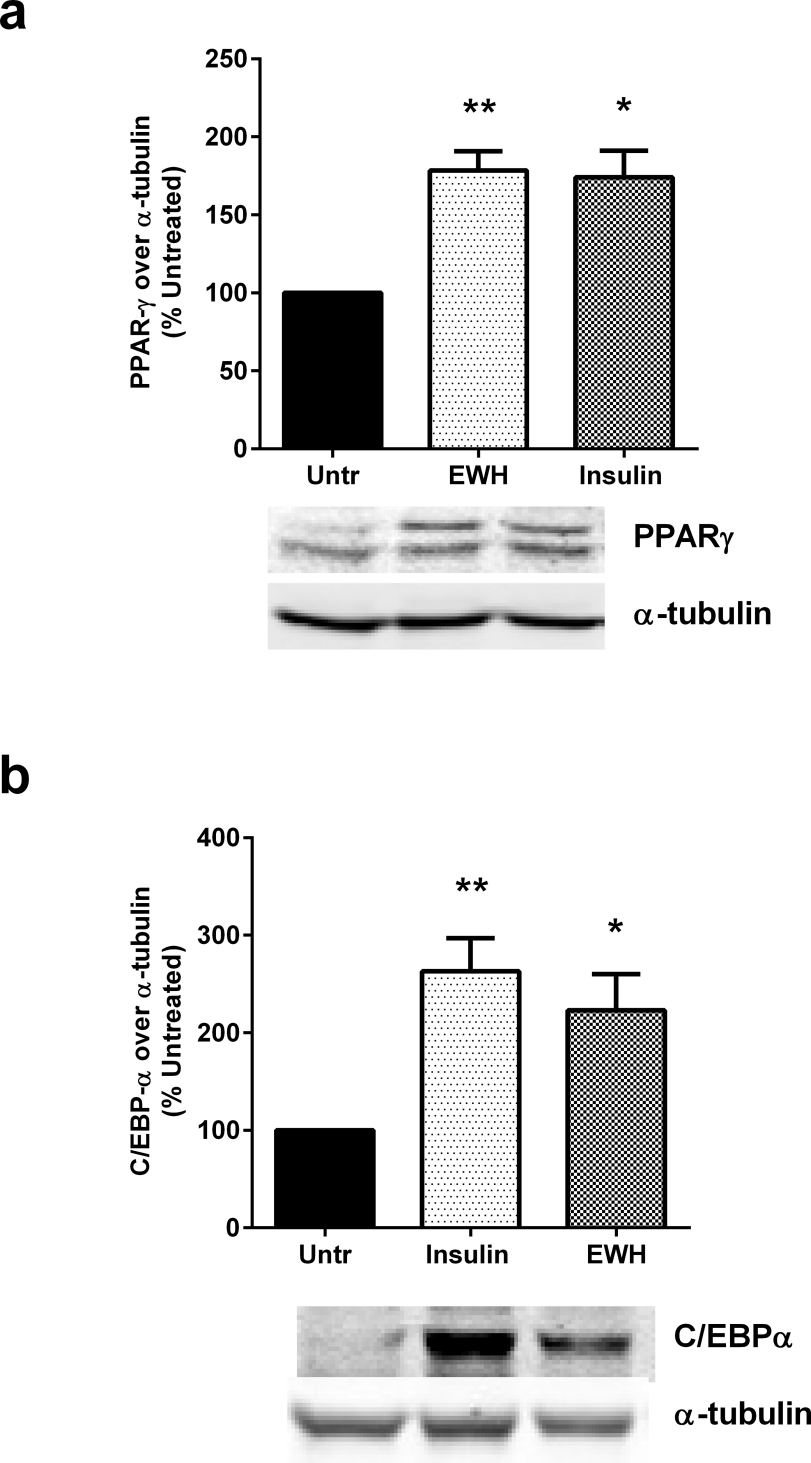
EWH upregulates markers of adipocyte differentiation. 3T3-F442A cells were incubated with EWH (5 mg/mL) or insulin (positive control; 10 μg/mL) for 72 hrs. The cells were then lysed and western blotting of the lysates was performed with antibodies against PPARγ (a), C/EBP-α (b) and α-tubulin (loading control; both a and b). A representative set of images are shown. Bands were quantified by densitometric analysis. Data are presented as mean±SEM of 4–5 independent experiments. *, ** and *** indicate p<0.05, p<0.01 and p<0.001 respectively, compared to the untreated control (Untr).

In addition to lipid accumulation and adiponectin release, adipocyte differentiation is also accompanied by increased expression of a number of proteins involved in different stages of this process. For example, PPARγ, an anti-inflammatory metabolic modulator is highly expressed in differentiated adipocytes and contributes to insulin sensitizing actions [37]. Similarly, C/EBP-α, a transcriptional regulator, is upregulated during differentiation where it co-ordinates the expression of downstream proteins involved in adipogenesis [35]. Indeed, 72 hrs incubation of 3T3-F442A cells with EWH upregulated both PPARγ (Fig 2A) and C/EBP-α (Fig 2B), demonstrating the successful induction of adipogenic differentiation event at the molecular level. Both effects were also comparable to those induced by insulin, the physiological agonist of adipocyte differentiation.

### EWH upregulates PPARγ expression dose-dependently in 3T3-F442A cells

**Fig 3:**
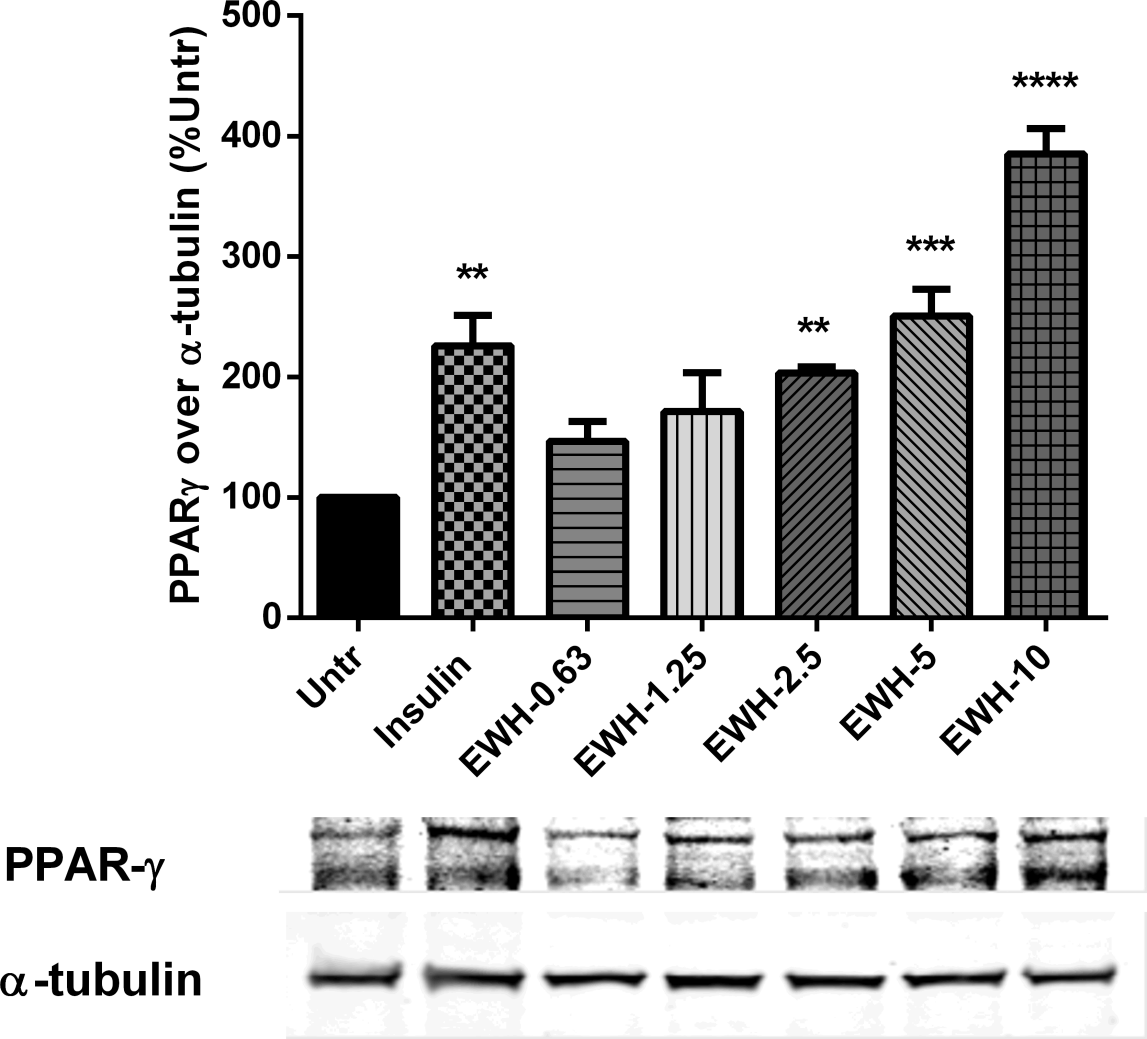
EWH upregulates PPARγ expression in a dose-dependent manner. 3T3-F442A cells were incubated with different dosages of EWH (0.63–10 mg/mL) or insulin (10 μg/mL) for 72 hrs. The cells were then lysed and western blotting of the lysates was performed with antibodies against PPARγ and α-tubulin (loading control). A representative set of images is shown. Bands were quantified by densitometric analysis. Data are presented as mean±SEM of 3–4 independent experiments. **, *** and **** indicate p<0.01, p<0.001 and p<0.0001 respectively, compared to the untreated control (Untr).

The effects of different concentrations of EWH on PPARγ expression was also investigated in 3T3-F442A cells. Fig 3 illustrates that EWH enhanced PPARγ expression in a dose-dependent manner. EWH at concentrations of 2.5, 5 and 10 mg/mL enhanced PPARγ expression significantly as compared to untreated cells at P<0.01, P<0.001 and P<0.0001 respectively.

### EWH exerts both insulin mimetic and insulin sensitizing effects

**Fig 4:**
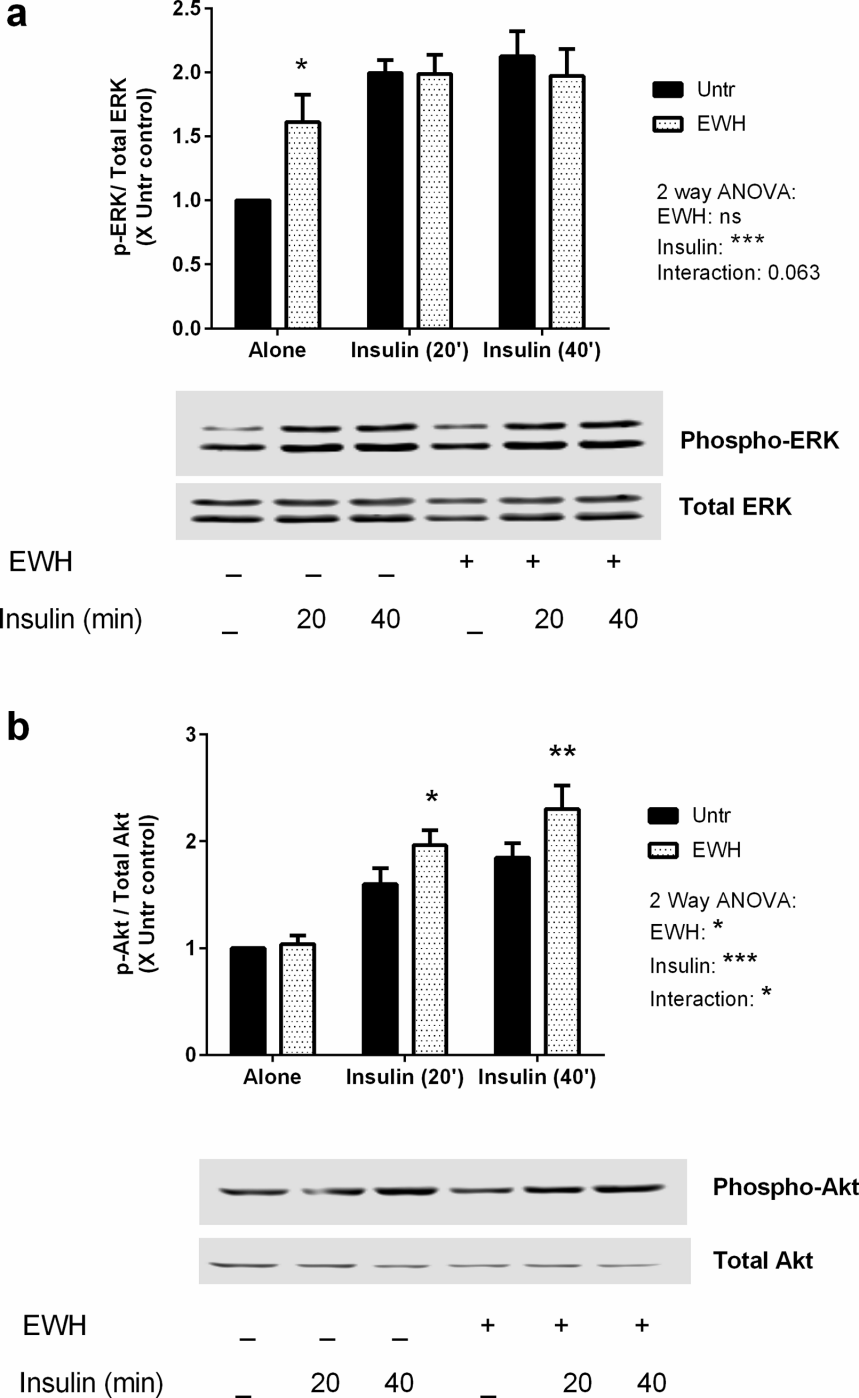
EWH differentially modulates insulin-mediated phosphorylation of ERK and Akt. 3T3-F442A cells were incubated with EWH (5 mg/mL) for 48 hrs prior to stimulation with insulin (10 μg/mL) for 20 or 40 min. The cells were then lysed and western blotting of the lysates was performed with antibodies against the total and phosphorylated forms of ERK (a) and Akt (b). Representative sets of images are shown. Bands were quantified by densitometric analysis. Data are presented as mean±SEM of 5 independent experiments. *, ** and *** indicate p<0.05, p<0.01 and p<0.001 respectively compared to untreated control by a two-way ANOVA. ‘ns’ indicates: not significant.

Given the similarity in EWH responses to insulin effects, we then investigated the effect of EWH on key insulin signaling pathways in these cells. The mitogen activated protein kinase ERK1/2 is an important downstream signaling target of insulin, which is phosphorylated (and hence, activated) by insulin treatment [38]. Treatment with EWH alone showed increased ERK1/2 phosphorylation in pre-adipocytes, while insulin-induced ERK1/2 activation in EWH-treated cells was comparable to that observed in EWH-free cells (Fig 4A), suggesting a potential insulin mimetic action of EWH.

Another major signaling target of insulin is protein kinase B (PKB)/Akt. Akt regulates many cellular processes including metabolism, proliferation, cell survival, growth and angiogenesis [39]. Akt phosphorylation is a key event involved in mediating the beneficial actions of insulin in glucose transport in adipose tissues [40]. Interestingly, EWH alone had no effects on Akt phosphorylation; while insulin actions on Akt phosphorylation were enhanced in EWH-treated cells over and above the response seen in control cells (Fig 4B). Indeed, a 2-way ANOVA showed a significant interaction between EWH and insulin effects on Akt phosphorylation indicating a novel insulin sensitizing action for EWH in these cells.

### EWH appears to involve insulin receptor signaling in adipocytes

**Fig 5:**
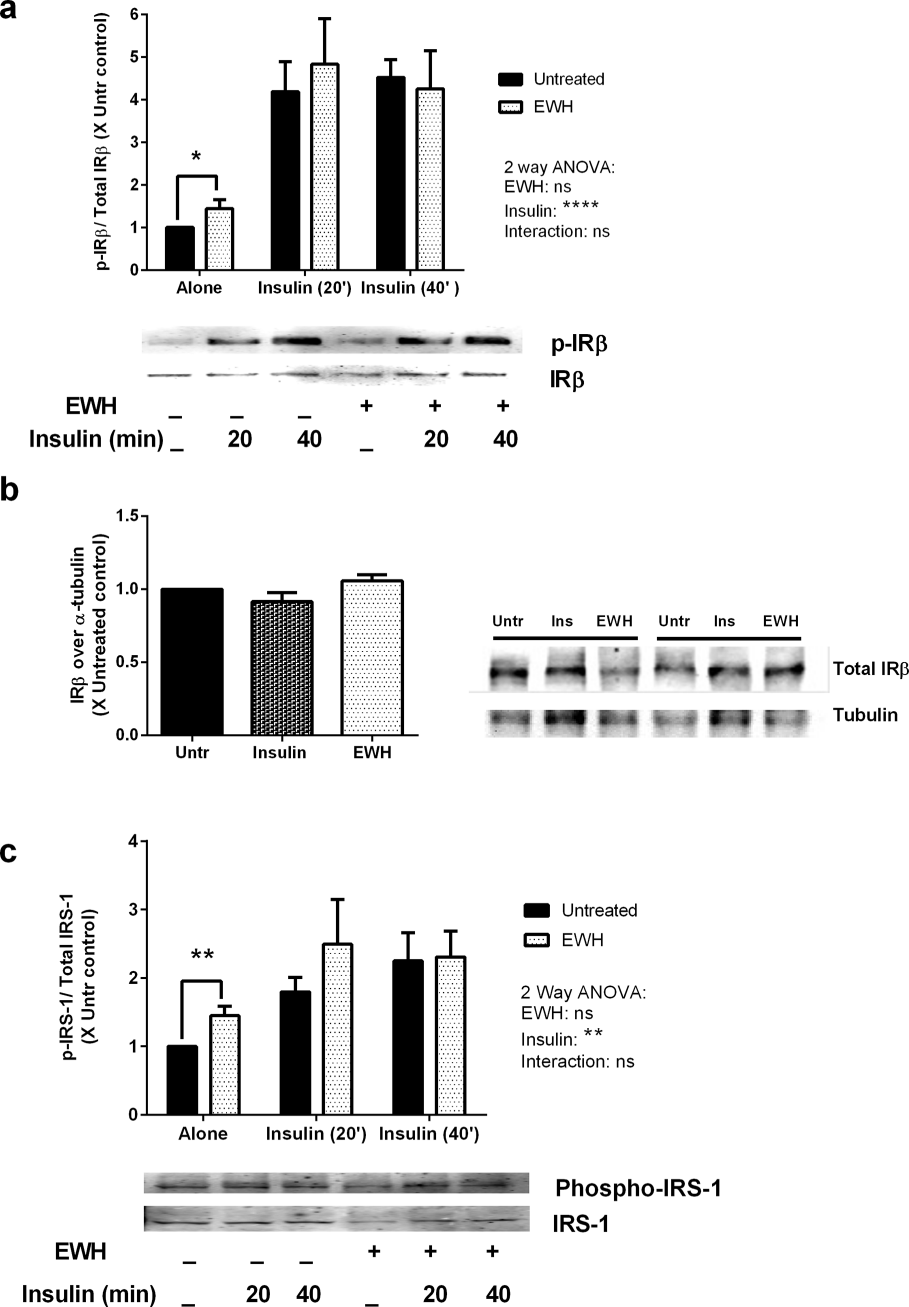
EWH actions are partly mediated through insulin receptor. 3T3-F442A cells were incubated with EWH (5 mg/mL) for 48 hrs prior to stimulation with insulin (10 μg/mL) for 20 or 40 min. The cells were then lysed and western blotting of the lysates was performed with antibodies against the: total and phosphorylated forms of IRβ (a), total IRβ and α-tubulin (loading control) (b), and total and phosphorylated forms of IRS-1 (c). Representative sets of images are shown. Bands were quantified by densitometric analysis. Data are presented as mean±SEM of 4–7 independent experiments. *, ** and **** indicate p<0.05, p<0.01, and p<0.0001 respectively.

Next, we examined if the observed EWH actions were dependent on signaling through the insulin receptor. Insulin binding to its receptor leads to phosphorylation of an associated protein, insulin receptor substrate 1 (IRS-1), which is widely used as a marker for insulin receptor mediated functions [41–43]. Interestingly, EWH treatment alone enhanced phosphorylation of insulin receptor β (IRβ) (Fig 5A, P<0.05), while the protein expression level of IRβ was not affected by the treatment (Fig 5B, P>0.05). Furthermore, EWH treatment alone also enhanced IRS-1 phosphorylation (Fig 5C) significantly (P<0.01) compared to untreated cells indicating potential insulin mimetic actions of EWH in these cells. However, EWH did not further enhance the phosphorylation of IRβ and IRS-1 in the presence of exogenous insulin. Only insulin was able to induce significant phosphorylation of IRβ and IRS-1, which remained unaffected by concomitant presence of EWH (Fig 5A and 5C).

### Insulin mimetic effect of EWH on ERK phosphorylation is mediated through insulin receptor in adipocytes

**Fig 6:**
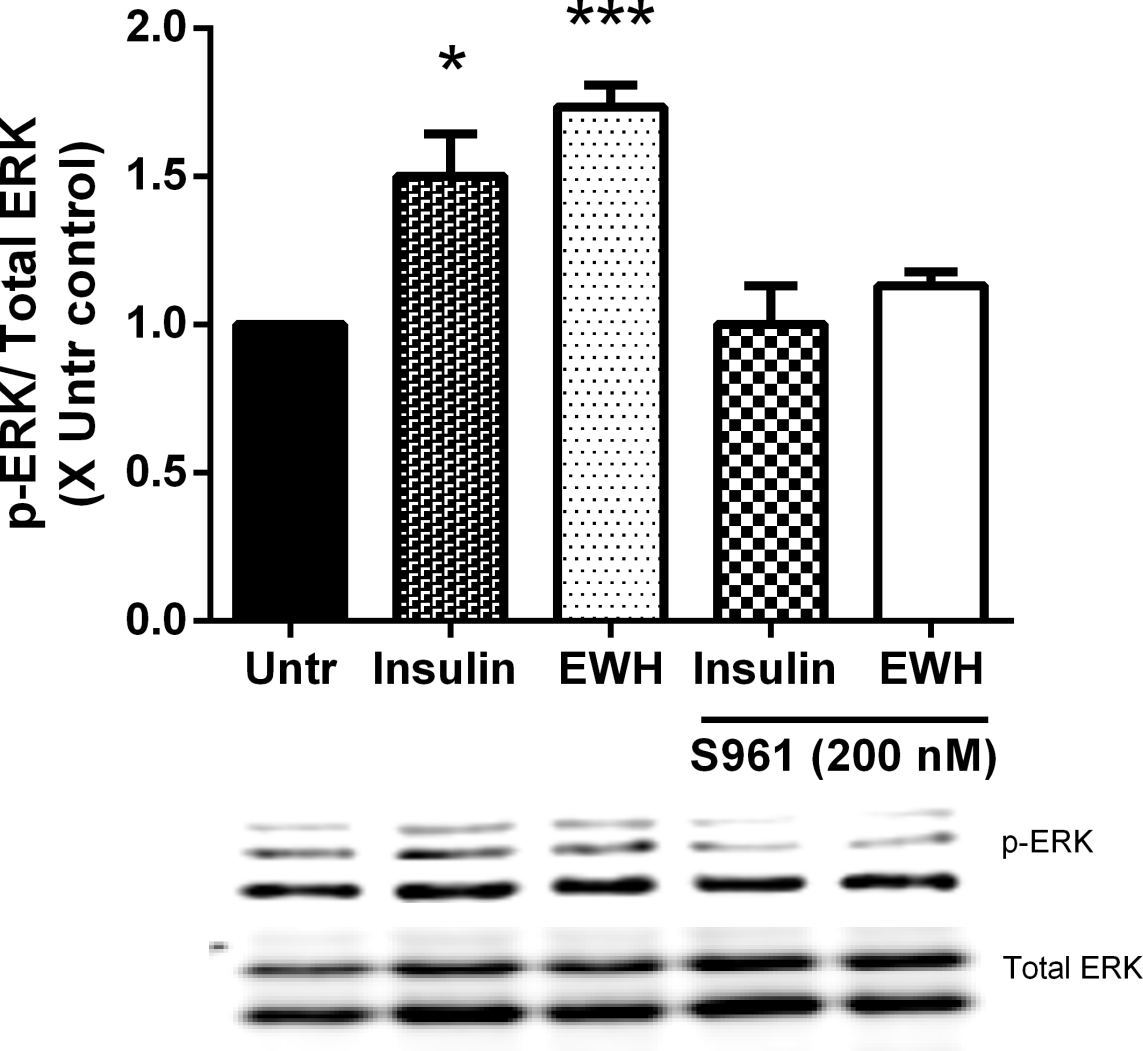
EWH mediates its insulin mimetic effects through insulin receptor. 3T3-F442A cells were incubated with EWH (5 mg/mL) or insulin (positive control; 10 μg/mL) in the presence/absence of S961 (insulin receptor antagonist; 200 nM) for 72 hrs. The cells were then lysed and western blotting of the lysates was performed with antibodies against the total and phosphorylated forms of ERK. A representative image is shown. Bands were quantified by densitometric analysis. Data are presented as mean±SEM of 3 independent experiments. *, and *** indicate p<0.05 and p<0.001 compared to untreated cells respectively.

Since EWH exerted insulin mimetic effects in 3T3-F442A cells (Fig 4A) and was mediated through insulin receptor (Fig 5A), we aimed to further explore this possibility by using S961, an insulin receptor antagonist. As indicated in Fig 6, EWH enhanced ERK phosphorylation significantly compared to untreated cells (P<0.001) (similar to insulin, P<0.05), whereas, incubating in the presence of the insulin receptor antagonist (S961) blocked the observed effects of both insulin and EWH on the levels comparable to the untreated control. This data suggests that, the insulin mimetic effects of EWH on ERK phosphorylation is potentially mediated through insulin receptor.

### EWH modulates inflammatory response in 3T3-F442A cells

**Fig 7:**
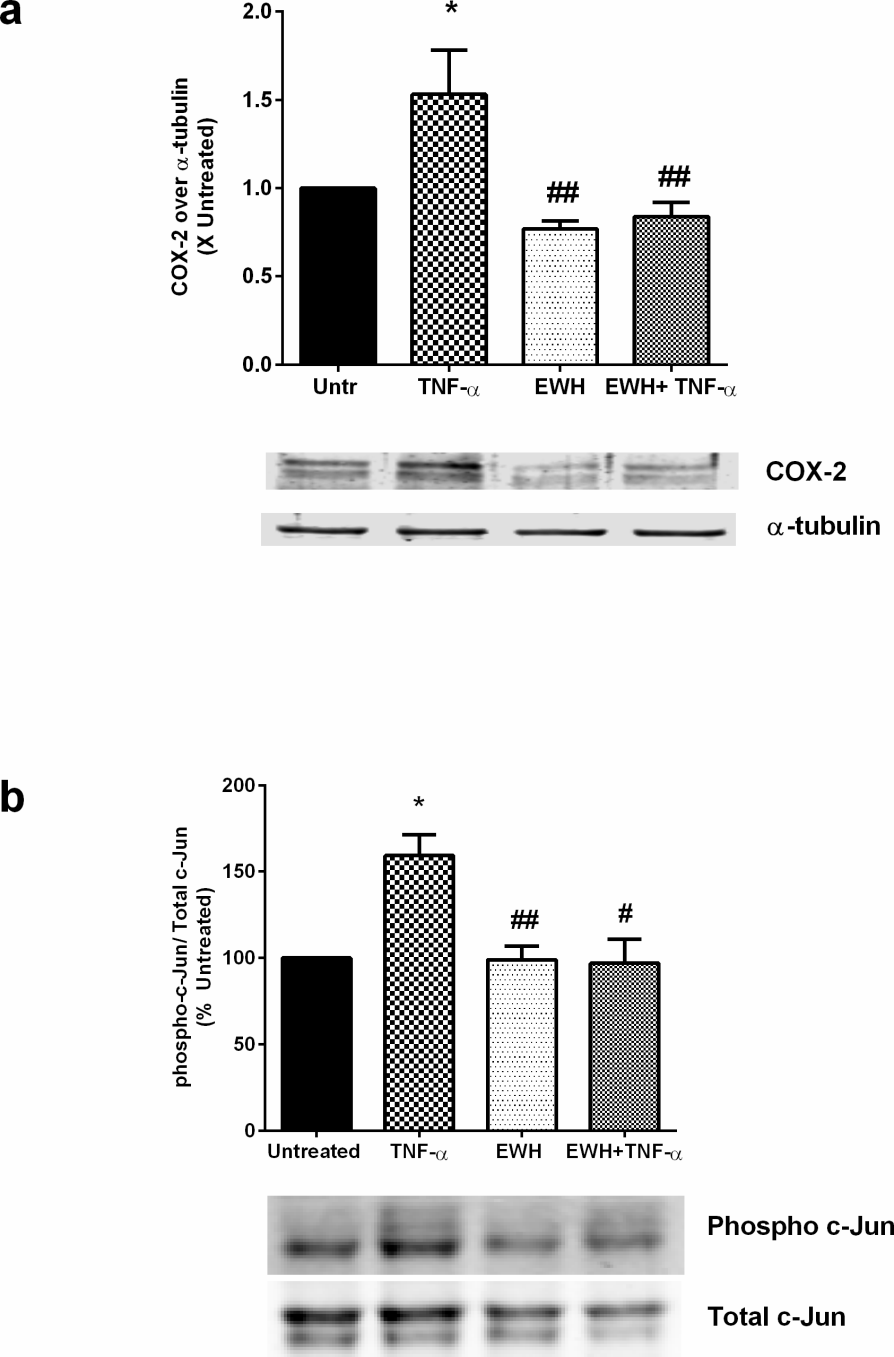
EWH exerts inhibitory effects on adipocyte inflammation. The 3T3-F442A cells were incubated with EWH (5 mg/mL) for 48 hrs prior to stimulation with TNF-α for either (a) 24 hrs (for inflammatory marker expression) or (b) 15 min (for transcription pathway experiment). The cells were then lysed and western blotting of the lysates was performed with antibodies against COX-2 and the loading control α-tubulin (a) or the total and phosphorylated forms of c-Jun (b). A representative set of images are shown. Bands from the COX-2 study were quantified by densitometric analysis. Data is mean±SEM of 4–6 independent experiments. * indicates p<0.05 compared to the untreated (Untr) group, while ## indicates p<0.01 compared to TNF-α alone.

Finally, we investigated the effects of EWH on inflammatory changes in these cells. Adipocyte inflammation releases harmful cytokines which leads to the loss of protective adipokines, increases insulin resistance and contributes to the pathogenesis of metabolic syndrome [44,45]. We used TNF-α, a pro-inflammatory cytokine involved in various inflammatory, atherosclerotic and metabolic disorders, to induce inflammation in these cells. Treatment with TNF-α for 24 hrs upregulated cyclooxygenase -2 (COX-2) levels in 3T3-F442A cells (1.64 ± 0.21), while a 48 hrs pre-treatment with EWH abolished this response (0.84 ± 0.19) (Fig 7A), indicating potentially beneficial anti-inflammatory capabilities of EWH. Further examination of underlying pro-inflammatory signaling/transcriptional pathways revealed a reduction of TNF-α-mediated c-Jun phosphorylation in EWH-treated cells. TNF-α increased c-Jun phosphorylation to159.20 ± 12.17% in adipocytes, while EWH treatment restored it to the basal level (98.15 ± 15.08%) (Fig 7B). This may account for the mechanisms underlying the anti-inflammatory actions of EWH.

## Discussion

Adipose tissue playing a vital role in energy homeostasis, is one of the critical target organs for insulin actions [11]. Adipose tissue secretes a number of adipokines interacting with central and peripheral organs in the body [10]. This insulin sensitive tissue influences diverse metabolic processes including carbohydrate metabolism, lipid metabolism, inflammation, blood pressure, energy expenditure, and feeding behavior [46,47]. In metabolically normal conditions adipocytes are small in size, sensitive to insulin and secrete insulin sensitizing hormones such as adiponectin [35]. In metabolic disorders on the contrary, adipocytes become larger in size, inflamed, insulin resistant, and increasingly express harmful adipokines leading to adipose tissue dysfunction, insulin resistance and associated diseases [14]. In fact loss of insulin sensitivity in adipose tissue adversely affects glucose utilization and lipid storage leading to ectopic deposition of fat in insulin sensitive tissues which contributes towards the development of insulin resistance and pathogenesis of both type II diabetes and metabolic syndrome [9,48]. Enhanced differentiation of fibroblast-like pre-adipocytes into mature adipocytes by the use of compounds mimicking insulin functions or enhancing insulin sensitivity provides a novel strategy for controlling the complications of metabolic syndrome [49–51].

PPAR*γ* and C/EBP-*α* are the two key molecules involved in adipocyte differentiation and regulation of the adipogenic network [52]. Over expression of PPARγ can induce adipogenesis in mouse embryonic fibroblasts lacking C/EBPα, but C/EBPα cannot rescue adipogenesis when PPARγ is not expressed, showing that PPARγ is the master regulator of adipogenesis [37,53,54]. Thiazolidinediones with insulin sensitizing effects promote pre-adipocytes differentiation by PPARγ activation [55,56]. Insulin also promotes adipogenic effects by upregulating both PPARγ and C/EBP-α in adipocytes [37,57]. Our study revealed that EWH treatment also exerted insulin-like differentiating effects on pre-adipocytes. In accordance with observed insulin-like effects of EWH on upregulation of pre-adipocyte differentiation markers, this treatment also enhanced PPAR*γ* and C/EBP-*α* to the same extent as insulin in adipocytes. So, it is plausible to propose that the observed insulin-like properties of EWH on adipogenic response may at least be in part due to involvement of these 2 regulators in 3T3-F442A cells. Indeed, several groups have identified plant derived novel compounds that promote adipogenic effects at least partially through upregulation of PPAR*γ* [58,59].

Binding of insulin to insulin receptor triggers the phosphorylation of IRβ and consequently, insulin receptor substrate (IRS) proteins providing the basis for the subsequent association with downstream signaling through different pathways mediating metabolic and mitogenic responses of insulin [42,60]. While phosphorylation and activation of Akt is responsible for most of the known metabolic effects of insulin, ERK phosphorylation mediates mitogenic and transcriptional effects of insulin in adipocytes [61]. When investigating the effects of EWH on insulin signaling, we also observed an insulin mimetic effect of EWH on ERK1/2 phosphorylation in these cells. In addition to the observed insulin mimetic effects on 3T3-F442A cells, EWH also exhibited insulin sensitizing effects by enhancing insulin-mediated Akt phosphorylation. Akt acts not only as a regulator of glucose transport but also involves in several other metabolic actions including glycolysis, protein synthesis, lipogenesis, glycogen synthesis, suppression of gluconeogenesis, cell survival, determination of cell size and cell-cycle progression [62]. The fact that EWH affected both ERK and Akt phosphorylation in adipocytes indicates the potential effects of this treatment on both pathways of insulin signaling.

Moreover, since phosphorylation of IRβ and IRS-1 was significantly enhanced in EWH treated cells while no further increase was observed in the presence of exogenous insulin suggesting that EWH exerts its insulin mimetic effects through insulin receptor which was further supported by the study of an insulin receptor antagonist (Fig 6). Therefore, the insulin sensitizing effect of EWH might be via targets downstream to IRS-1 such as phosphoinositide-3 kinase (PI3-kinase) and phosphoinositide-dependent kinase 1 (PDK1).

There is an interest to assess the potential of established antihypertensive compounds for protection against insulin resistance and other complications of metabolic syndrome due to the role of RAS impairment in the pathogenesis of such diseases [63]. RAS blockade has been reported to inhibit the body fat mass increase [64,65], and improve insulin resistance and glucose tolerance in type-2 diabetic rodents [66,67]. Captopril, the pharmacological ACE inhibitor, has been reported to enhance adipocyte differentiation and reduce inflammation in various tissues [68]. Insulin sensitizing effects of RAS blockade have also been reported in clinical studies in patients with risk factors [69–71] suggesting additional benefits of these drugs in a complex condition like metabolic syndrome. Interestingly, the anti-hypertensive EWH with RAS modulating properties (reducing vascular ACE and angiotenstin II type 1 receptor expression) also enhanced pre-adipocyte differentiation, and induced insulin mimetic and sensitizing effects in 3T3-F442A cells. Similarly, milk derived peptides IPP and VPP, with ACE-inhibitory and anti-inflammatory properties exerted insulin mimetic adipogenic effects by promoting the differentiating of pre-adipocytes in 3T3-F442A cells [33]. The flaxseed protein hydrolysate contains peptide fractions with anti-hypertensive [72] as well as anti-diabetic properties [73]. Since hypertension, inflammation and insulin resistance present concomitantly in many cases of metabolic syndrome, EWH as a novel naturally based compound with multiple benefits against hypertension, inflammation and insulin functions may serve as an effective option for the management of complications of this disease.

Adipose tissue inflammation with dysregulated adipokine secretion plays a critical role in the development of a variety of cardiometabolic disorders including metabolic syndrome, type 2 diabetes, inflammatory and vascular disorders and eventually development of coronary heart disease [9]. EWH upregulated the expression of anti-inflammatory molecules such as PPARγ and adiponectin, demonstrating the potential benefits of EWH on adipocyte function and metabolic syndrome. EWH also prevented the TNF-α-mediated induction of the pro-inflammatory enzyme COX-2, a molecule that contributes to the pathologic complications of metabolic syndrome [74,75]. This anti-inflammatory effect is likely due to its interference with the AP-1 transcription factor pathway which is involved in COX-2 expression and can be modulated by the inhibition of c-Jun phosphorylation [76–78]. TNF-α stimulates the pro-inflammatory phenotype in adipose tissues leading to the development of insulin resistance and metabolic syndrome [11]. Indeed, suppression of TNF-α has been suggested as a potential therapy against metabolic syndrome [79]. VPP and IPP prevented inflammatory changes in 3T3-F442A cells [33]. In another study, Sawada et al. have reported that VPP inhibited adipose inflammation *in vitro* and *in vivo* [80]. VPP also enhanced insulin sensitivity in obese mice and inhibited macrophage accumulation and activation in fat tissues [80]. Moreover, beta-mercaptoethanol (BME), the pharmacological redox regulator and radical scavenger, has also been reported to down-regulate the expression of inflammatory cytokines and promote adipocyte differentiation [81]. Our data with EWH further supports its role as a novel regulator of adipose functions with additional anti-inflammatory benefits.

Bioactive peptides in the EWH are potentially responsible for the observed effects of EWH on adipogenic differentiation, insulin signaling and anti-inflammatory responses in adipocytes. Purification and fractionation of EWH to identify its responsible peptides with beneficial effects on adipocyte differentiation is essential in understanding the structure requirements of food-derived bioactive peptides with beneficial effects on adipose tissue function. We have fractionated EWH using stepwise chromatographic methods and have characterized the peptides responsible for adipogenic responses in adipocytes (PPARγ expression). Among total 42 peptides identified from EWH, of four peptides (ERYPIL, VFKGL, WEKAFKDED, and QAMPFRVTEQE) significantly enhanced PPARγ expression, compared to untreated cells (*unpublished data*).

In conclusion, our study demonstrated that EWH, with RAS modulating properties promoted adipocyte differentiation through a combination of insulin mimetic and insulin sensitizing actions on 3T3-F442A cells. In addition, EWH also increased expression of the anti-inflammatory hormone adiponectin and suppressed cytokine mediated inflammatory response in these cells. Considering the fundamental role of adipose tissue dysfunction in the pathogenesis of hypertension, inflammation, insulin resistance, and metabolic syndrome, EWH may have potential benefits in the prevention and management of metabolic syndrome.

## Supporting information

S1 File The relevant data used for generating figures and statistical analysis in this study. (XLSX)
